# Case Report: CAR-T therapy for primary cerebellar ALK-negative anaplastic large cell lymphoma

**DOI:** 10.3389/fimmu.2025.1570214

**Published:** 2025-07-24

**Authors:** Zhongqing Zou, Sihan Lai, Hai Yi, Qian Zhang, Hao Yao, Hui Yang, Ling Zhang, Alex H. Chang, Yi Su, Jin Rao

**Affiliations:** ^1^ Department of Hematology, Clinical Medical College & Affiliated Hospital of Chengdu University, Chengdu University, Chengdu, China; ^2^ Department of Hematology, The General Hospital of Western Theater Command, Sichuan Clinical Research Center for Hematological Disease, Branch of National Clinical Research Center for Hematological Disease, Chengdu, China; ^3^ Engineering Research Center of Gene Technology, Ministry of Education, Institute of Genetics, School of Life Sciences, Fudan University, Shanghai, China; ^4^ Shanghai YaKe Biotechnology Ltd., Shanghai, China

**Keywords:** primary central nervous system lymphoma (PCNSL), T-cell lymphoma, cerebellar, ALK, ALCL, CAR-T, CD30, CD7

## Abstract

Primary central nervous system (CNS) T-cell lymphomas, such as anaplastic large-cell lymphoma (ALCL) with anaplastic lymphoma kinase (ALK) negativity, are relatively rare and aggressive. Despite the administration of high-dose methotrexate (HD-MTX) therapy, the prognosis remains pessimistic. We reported a 21-year-old male patient initially presented with headache and fever. Following surgical intervention, the pathological examination confirmed the diagnosis of ALK-negative ALCL, and the patient was in a critical postoperative condition, evidenced by a Glasgow Coma Scale (GCS) score ranging from 5 to 9. After undergoing chemotherapy with HD-MTX, CD30 and CD7-targeted chimeric antigen receptor T (CAR-T) cells were sequentially infused. The initial infusion dosage was 0.5×10^6^ cells per kilogram (kg) of body weight, which was subsequently adjusted to 1×10^6^ cells per kg. During this therapeutic process, grade 2 cytokine release syndrome (CRS) occurred. However, the CAR-T cells exhibited robust expansion in the peripheral blood, and the patient’s level of consciousness improved significantly, as indicated by an increase in the GCS score to a range of 12 to 15. A 15-month follow-up assessment revealed no evidence of tumor recurrence, and the patient was able to ambulate with the assistance of rehabilitation equipment. This case represents the first globally successful instance of treating ALK-negative primary central nervous system anaplastic large-cell lymphoma (PCNSALCL) with CAR-T therapy. It validates the feasibility and safety of the sequential CD30/CD7 CAR-T therapy for rare CNS T-cell lymphomas, offering a novel treatment strategy for these conditions. Nevertheless, further long-term follow-up and evaluation are essential to determine its sustained efficacy and immunological implications.

## Introduction

PCNSL is a rare type of extranodal non-Hodgkin’s lymphoma. Its lesions are limited to the brain parenchyma, spinal cord, leptomeninges, and eyes. More than 95% of PCNSL patients have diffuse large B-cell lymphoma as the pathological type. T-cell lymphoma, in particular ALK-negative ALCL, is highly infrequent in clinical practice (1–4). Combined therapy based on HD-MTX is currently the first-line treatment option for patients with PCNSL (3, 4). Although the treatment methods have been continuously advancing, the survival rate of PCNSL has merely shown a marginal improvement and the prognosis is still poor, especially for elderly patients. According to statistics, the 5-year survival rates stand at only 30% to 40% (5). Primary central nervous system T-cell lymphoma (PCNSTCL) is a rare disease, and large-sample studies on this tumor are extremely scarce. As far as we know, the current study with a relatively large sample size comes from the analysis of 59 patients with PCNSTCL registered in the SEER database. Although HD-MTX based regimens can improve the survival of patients with PCNSTCL, the median survival is only 8 months (5).

CAR-T cell therapy has revolutionized the treatment paradigm of hematological malignancies (6). Specifically, CAR-T cell therapy targeting CD19 has demonstrated favorable efficacy in both relapsed or refractory PCNSL and secondary central nervous system lymphoma (SCNSL) (7–9). While CAR-T cell therapy has exhibited remarkable safety and efficacy in the treatment regimen of B-cell-derived malignancies, it encounters more conspicuous limitations when applied to T-cell malignancies, specifically, concerns such as fratricide, wherein CAR-T cells may target normal T cells, T-cell dysplasia, and tumor cell contamination emerge (10). These limitations predominantly derive from the resemblance between normal T cells and malignant T cells, exacerbated by the dearth of a precisely defined and specific malignant T antigen. Nevertheless, certain targeting CD30 and CD7 CAR-T cell therapies have yielded rather promising treatment outcomes in T-cell lymphoma (11–13), whose effectiveness provides a glimmer of optimism within the otherwise arduous terrain of T-cell malignancy treatment and justifies further exploration and optimization.

We reported a case of newly diagnosed primary central nervous system ALK-negative ALCL located in the cerebellum. The patient’s post-operative state was precarious, on the verge of mortality, as shown by a GCS of 5-9, implying significant neurodeficit and attenuated awareness. After the implementation of chemotherapy based on HD-MTX, the patient underwent autologous sequential targeting CD30 and CD7 CAR-T therapies, with the intention of evaluating the efficacy and safety parameters of this therapeutic protocol. Our report indicates that CAR-T cell therapy could offer a novel treatment approach for PCNSTL patients, particularly those with the ALK-negative ALCL subtype.

## Materials and methods

### Ethical approval

The studies involving humans were approved by the Ethics Committee of the General Hospital of Western Theater Command, People’s Liberation Army, in accordance with the Declaration of Helsinki.

Consent documentation Participants or their legal guardians are required to execute a written informed consent document. This document comprehensively delineates the following critical components: potential risks and benefits associated with the study, available alternative therapeutic modalities, specifications for data anonymization and long-term follow-up protocols, the entitlement to withdraw from the study, and the corresponding withdrawal procedures.

### Data collection procedures

Participants provided written informed consent for the publication of potentially identifiable images and data. Baseline data, including demographic information, disease characteristics, laboratory indices, imaging findings, treatment-related data, and follow-up results, were systematically collected.

### CAR-T cell manufacturing method

Peripheral blood lymphocytes are collected from the patient, transduced with viral vectors encoding chimeric antigen receptor (CAR) genes targeting specific antigens (CD30/CD7), and then cultured and expanded *in vitro*, with rigorous quality control implemented throughout the entire process.

### Monitoring and toxicity grading

The grading criteria for CRS and Immune Effector Cell-Associated Neurotoxicity Syndrome (ICANS) are derived from the standardized guidelines of the American Society for Transplantation and Cellular Therapy (ASTCT).

## Results/case presentation

### Initial presentation

**Figure 1:**
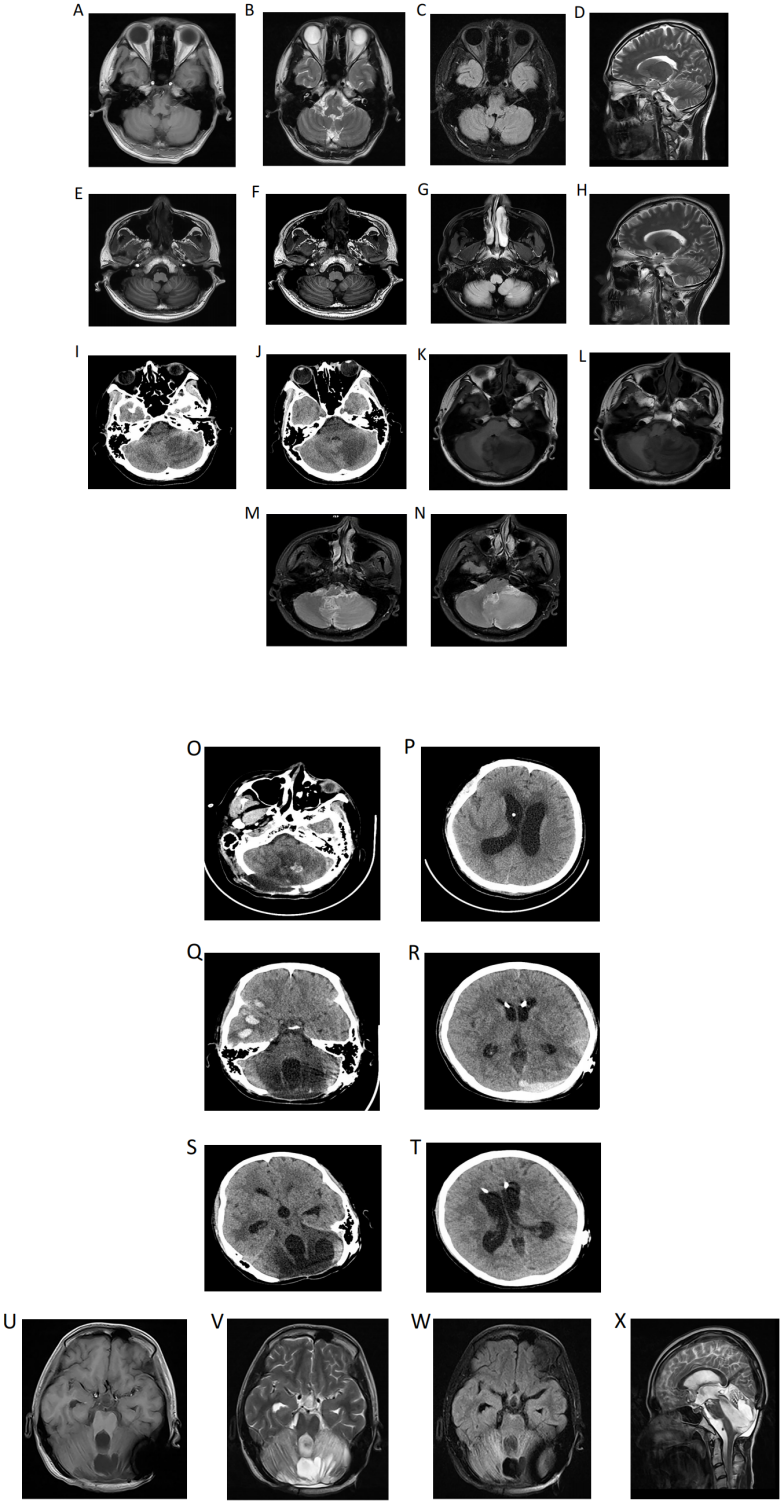
**(A)** (Diagnostic imaging): At the beginning of the onset of the disease, the cranial Magnetic Resonance Imaging (MRI) demonstrated no evident intracranial abnormalities, and the cranial diffusion-weighted imaging (DWI) indicated the absence of newly developed cerebral infarction **(A-D)**. Following headache exacerbation and recurrent fevers, the repeat cranial MRI demonstrated the presence of patchy slightly hyperintense signals on both T1-weighted **(E, F)** and T2-weighted **(H)** images within the white matter of the left cerebellar hemisphere, with hyperintense signals on fluid-attenuated inversion recovery (FLAIR) **(G)**. While the cranial computed tomography (CT) demonstrated a slightly low-density shadow in the left cerebellar hemisphere. The etiological determination remains elusive. While an inflammatory or infectious origin is one of the considerations, other differential diagnoses must also be discussed. Notably, the anatomical configuration and spatial dimensions of each ventricle and cistern were observed to be within the parameters of normality **(I, J)**. The headache worsens further, at this time, in the left cerebellar hemisphere, middle cerebellar peduncle and cerebellar vermis, extensive regions with poorly delineated boundaries manifest as slightly long T1 **(K, L)** and T2 **(M, N)** signal shadows, which further present as slightly high signals on FLAIR. **(B)** (Follow-up imaging): Post-operative changes in the cerebellum: cerebellar tissue swelling with scattered punctate hemorrhages **(O, P)**. Multiple contusions and lacerations accompanied by hematoma formation in the right frontal and temporal lobes, epidural hematoma in the left occipital region **(Q, R)**. CT manifestations of right temporal lobe hematoma and left occipital epidural hematoma in the absorption stage **(S, T)**. Partial defects are found in the cerebellar tissue and occipital bone. In the center of the remaining cerebellar tissue, there are mixed high and low signals, mainly long T1 and long T2 signals, and the signal of the surrounding cerebellar parenchyma is significantly increased on T2WI **(U-X)**.

A 21-year-old male patient was admitted to the Department of Neurology in our hospital on September 11, 2023, presenting with a one-week history of cough, fever, and headache. In light of the positive neck impedance, Kernig’s sign and Brudzinski’s sign, a lumbar puncture was performed. Cerebrospinal Fluid (CSF) pressure was measured at 240 mmH_2_O, CSF examination demonstrated an elevated white blood cell count of 154×10^6^/L with 40% polymorphonuclear nucleated cells and 60% mononuclear nucleated cells, a mildly elevated protein level at 0.73 g/L, and the absence of acid-fast bacilli, cryptococcus, and bacteria. The cranial Magnetic Resonance Imaging (MRI) along with diffusion-weighted imaging (DWI) revealed no conspicuous intracranial abnormalities (**Figures 1A-D**). Based on the above-mentioned evidence, viral encephalitis was preliminarily considered and treated with acyclovir and dexamethasone for 13 days, during which the patient’s headache was partly relieved but the fever persisted. A repeat CSF examination revealed nucleated cell count 130×10^6^/L, microprotein 0.87 g/L, glucose 2.31 mmol/L, chlorine 116 mmol/L, while the CSF smear showed no conspicuous pathological alterations. In the CSF total bile acid detection, positive signals were seen in the hippocampal zone featuring staining in neuronal cells of the corpus callosum, and also in the cerebellar region with staining in the medulla. The repeat cranial MRI demonstrated the differential diagnosis encompassed inflammatory or infectious pathologies, along with other potential etiologies (**Figures 1E-H**), accordingly, meningoencephalitis was also considered, especially regarding its viral, tuberculous or immunological origin. The patient underwent a comprehensive treatment involving acyclovir, meropenem, gamma globulin, and a diagnostic treatment combination of rifampicin, isoniazid, ethambutol and pyrazinamide. Consequently, his fever remitted and the symptoms of dizziness and cephalalgia alleviated. Subsequently, the patient was discharged on October 11, 2023.

### Surgical interventions

Unfortunately, ten days post-discharge, the patient suffered from exacerbated headache. Subsequently, the emergency cranial CT on October 25, 2023 demonstrated a slightly low-density shadow in the left cerebellar hemisphere (**Figures 1I, J**). The quadruple diagnostic anti-tuberculosis drugs were adjusted to (rifampicin, isoniazid, moxifloxacin, pyrazinamide). Besides, linezolid, meropenem and sulfamethoxazole were used for anti-infective treatment, however, he still suffered from recurrent fevers and aggravated headache. On October 29, 2023, the cranial MRI was performed and a neurosurgery consult was requested. A left cerebellar space-occupying lesion resection and decompressive craniectomy were emergently carried out under general anesthesia as a possible foramen magnum hernia was considered (**Figures 1K-N**), and the operation went smoothly. Subsequently, the patient was sent to the intensive care unit (ICU). Once there, the endotracheal tube was removed, and then he was taken off the ventilator and regained consciousness. Disappointingly, on November 8, 2023, the patient’s consciousness disturbance worsened, with occasional bilateral pupil size disparity and sluggish light reflex. Immediately, an emergency right cerebellar space-occupying lesion resection and decompressive craniectomy were done under general anesthesia. Postoperatively, the patient was transferred to the ICU for ongoing care but remained comatose (**Figures 1O, P**).

### Pathological diagnosis and subsequent treatment

**Figure 2:**
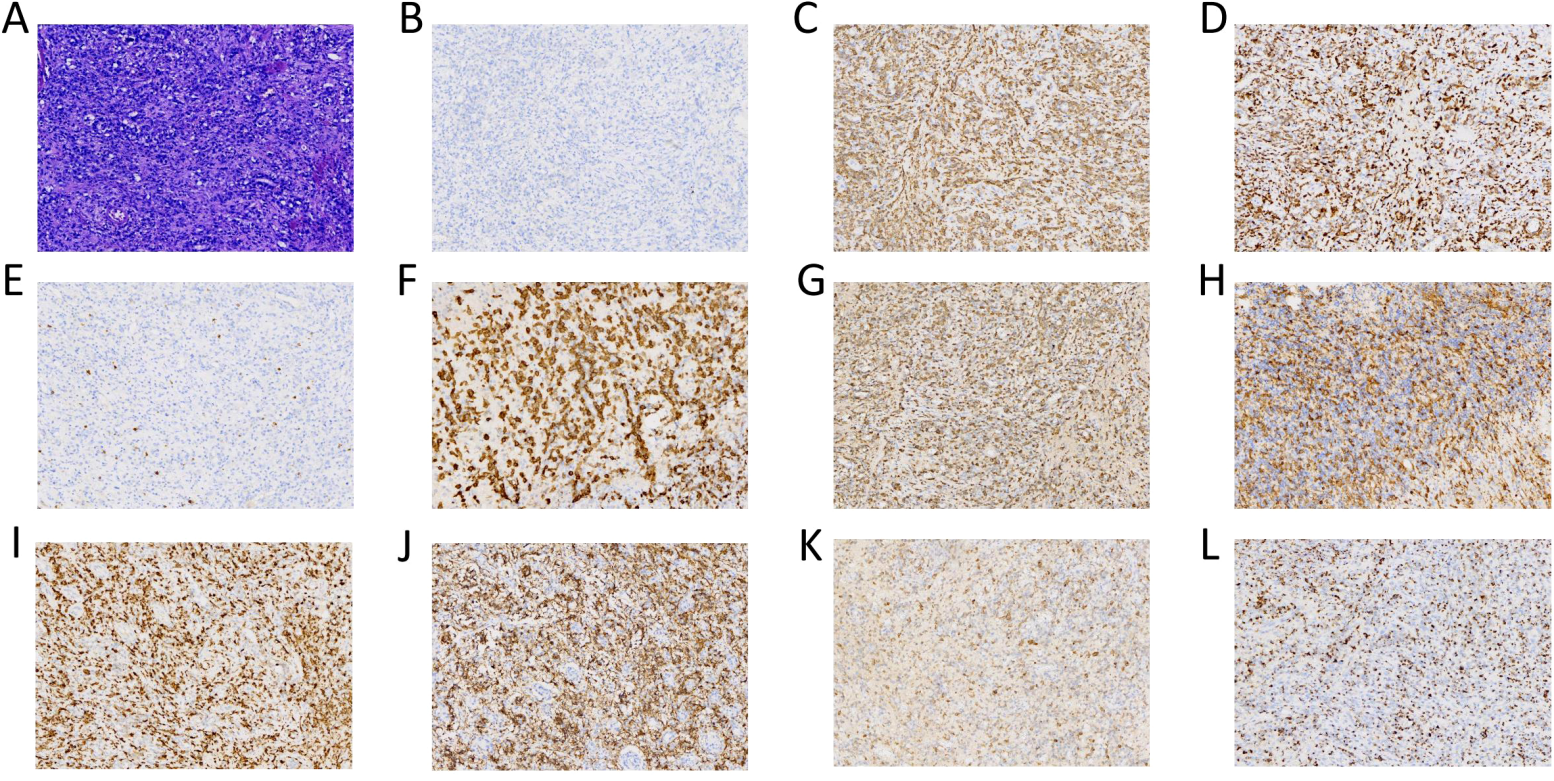
(Histopathology): The post-operative cerebellar pathological diagnosis is cytotoxic T- cell lymphoma, and considered as anaplastic large cell lymphoma. The histological morphology revealed a subset of these cells showed enlarged dimensions, with visible nuclear mitoses and significant vascular hyperplasia [**(A)** 20x]. The immunohistochemistry indicated: ALK negative [**(B)** 20x], CD2 positive [**(C)** 20x], CD3 positive [**(D)** 20x], CD5 negative [**(E)** 20x], CD30 positive [**(F)** 20x], GrB positive [**(G)** 20x], CD4 positive [**(H)** 20x], CD7 positive [**(I)** 20x], CD56 positive [**(J)** 20x], EMA positive [**(K)** 20x] and TIA-1 positive [**(L)** 20x].

**Figure 3:**
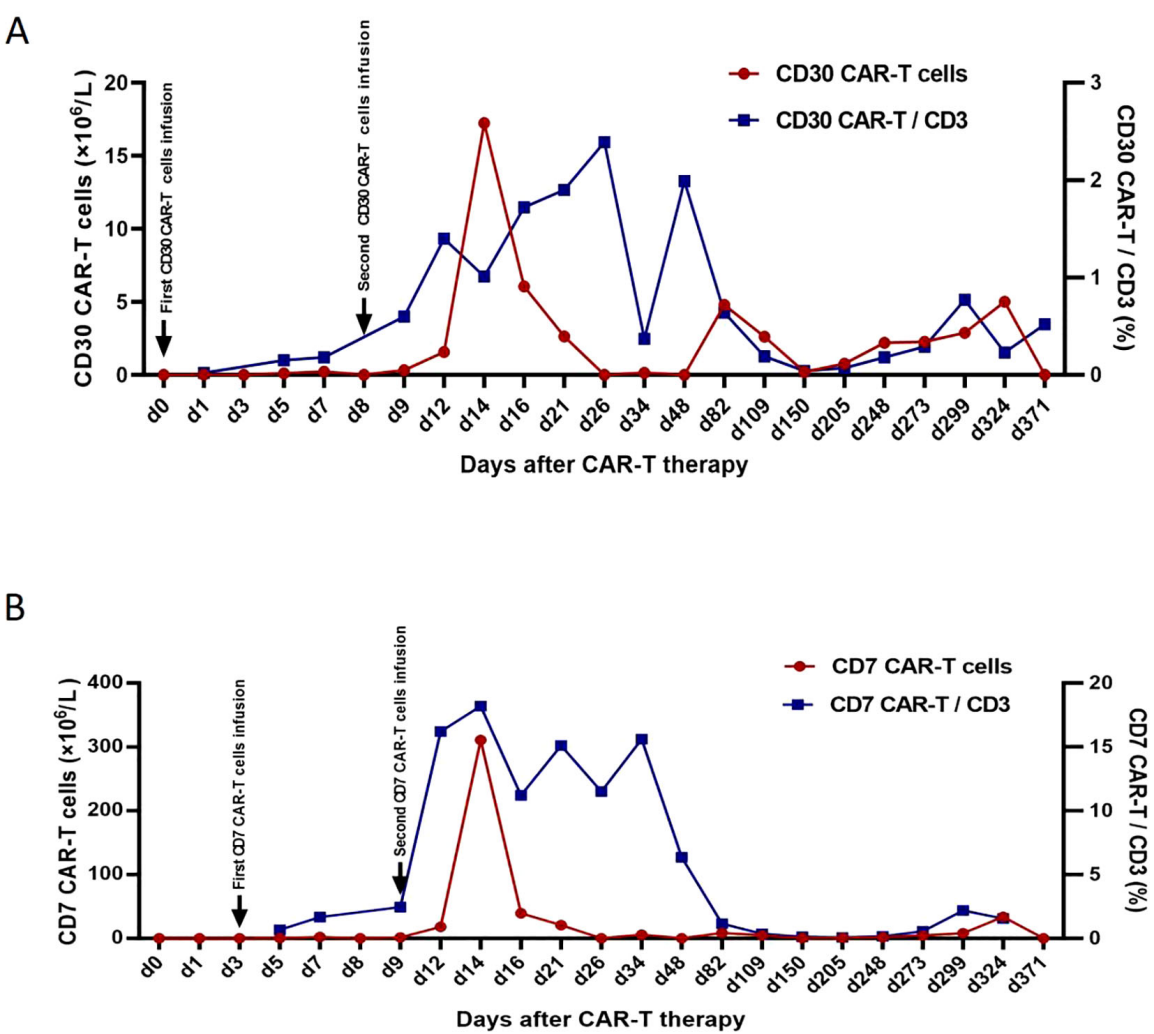
CD30-CAR-T cells from peripheral blood **(A)**, CD7-CAR-T cells from peripheral blood **(B)**. CAR, chimeric antigen receptor.

Postoperative pathology of the left cerebellum was considered ALCL with ALK negativity (**Figure 2**). Key clinical events timeline as shown in **Table 1**. Three days post-second surgery, the patient’s postoperative GCS score was 9. Compassionately, a combination chemotherapy with HD-MTX as the cornerstone was administered. Subsequently, lymphocytes were successfully collected, followed by the administration of the fludarabine-cyclophosphamide regimen for lymphocyte depletion. After that, CD30 and CD7 CAR-T cells at a dose of 0.5×10^6^/kg were sequentially infused smoothly on November 29 and December 2, 2023 respectively. However, peripheral blood (PB) monitoring revealed suboptimal expansion of both CD30 and CD7 CAR-T cells, subsequently, the second infusion of CD30 and CD7 CAR-T cells at a dose of 1×10^6^/kg were conducted without incident on December 7 and 8, 2023 respectively, though accompanied by grade 2 CRS. Notably, favorable expansion of the CAR-T cells was detected peripherally. Prior to the initiation of CAR-T therapy, the patient exhibited a fluctuating GCS score, ranging from 5 to 9. After the CAR-T treatment, the patient’s consciousness showed a notable improvement, as evidenced by the GCS score of 12 to 15. The peak numbers of CD30- and CD7-CAR-T per μL of PB were 17.24 on the 6th day and 310.66 on the 5th day after the second infusion respectively (**Figures 3A, B**). Following CAR-T treatment, the patient continued to receive anti-infection supportive treatment and airway management in the ICU ward, and one month post CAR-T therapy, the patient experienced a brief agranulocytosis episode that improved with anti-infection supportive treatment. Subsequently, the patient regained consciousness progressively and was transferred to the Department of Neurosurgery to receive a full course of antibacterial and antiviral treatments on March 15, 2024. When hydrocephalus was most evident, a ventriculoperitoneal shunt was performed. After approximately three months in the Department of Neurosurgery, the patient’s infection was under control and the general condition was satisfactory. On June 6, 2024, the patient was transferred to the Rehabilitation Department for further rehabilitation treatment. Chidamide was administered for maintenance treatment.

**Table 1:** Brief chronology of the key clinical events in this case.

Time before and after Chimeric Antigen Receptor T (CAR-T) cell therapy/d	Date	Key events
-79	2023.09.11	A one-week history of cough, fever, and headache.
-31	2023.10.29	Resection of left cerebellar space-occupying lesion with decompressive craniectomy.
-21	2023.11.08	Resection of right cerebellar space-occupying lesion with decompressive craniectomy.
-19	2023.11.10	Postoperative pathology of the left cerebellum indicated anaplastic large cell lymphoma (ALCL), anaplastic lymphoma kinase (ALK) negative; cyclophosphamide 300 mg + dexamethasone 5 mg was administered.
-18	2023.11.11	Selinexor 40 mg once weekly combined with high dose methotrexate 3.5 g/m² for chemotherapy.
-15	2023.11.14	Autologous lymphocyte collection was performed, and CAR-T cell preparation was initiated.
-11	2023.11.18	Thiotepa 50 mg for chemotherapy.
-7	2023.11.22	Intrathecal administration via ventricular drainage tube: methotrexate 15 mg + cytarabine 30 mg + dexamethasone 5 mg for central nervous system (CNS) chemotherapy.
-4	2023.11.25	Fludarabine 30 mg/m² on days 1 to 3 + cyclophosphamide 400 mg/m² on days 1 to 3.
0	2023.11.29	CD30-CAR-T cells: 0.5×10^6^/kg.
+3	2023.12.02	CD7-CAR-T cells: 0.5×10^6^/kg.
+8	2023.12.07	CD30-CAR-T cells: 1.0×10^6^/kg.
+9	2023.12.08	CD7-CAR-T cells: 1.0×10^6^/kg.
+190	2024.06.06	Chidamide 20 mg twice weekly for maintenance treatment (adjusted based on blood routine findings).
+306	2024.09.30	Cranial Magnetic Resonance Imaging (MRI) re-evaluation showed no evidence of tumor recurrence. The patient achieved independent rehabilitation with assistive devices and regained ambulatory function.

### Follow-up

Currently, the patient is able to conduct rehabilitation with the aid of equipment and has regained the ability to walk. Nevertheless, on October 4, 2024, the patient endured another fall, which led to cerebral parenchymal hemorrhage (**Figures 1Q, R**). Fortunately, surgical intervention was not required. After approximately one month of conservative management, the cerebral hemorrhage was progressively absorbed (**Figures 1S, T**). At this juncture, the patient still persists with rehabilitation treatment. Recently, the patient underwent a reexamination via cranial MRI (**Figures 1U-X**), and no evidence of recurrence was observable in relation to the cerebellar tumor.

## Discussion

PCNSALCL, being a T-cell lymphoma typified by the proliferation of large lymphoid cells expressing copious amounts of CD30 on the cell surface, constitutes an exceptionally scarce histological subtype (14–18). The median age at onset is 21 years (range, 1–82 years), with a preponderance of male cases (14). ALCL is classified into anaplastic lymphoma kinase ALK-positive ALCL and ALK-negative ALCL in accordance with ALK expression (19). Yudai Hirano et al. (14)reviewed the clinical features and prognostic factors of PCNSALCL. The median age was 63 years in the ALK-negative group, while it was 17.5 years in the ALK-positive group. The ALK-negative group exhibited an inferior prognostic profile, with a 2-year survival rate of 22% and a median survival time of 0.21 years, in comparison to the ALK-positive group which had a 2-year survival rate of 71%. Among the cohort of the 39 patients, within the framework of the cerebellum as the locus of disease origination, a 22-year-old ALK-negative female, in the absence of MTX intervention, demonstrated a survival period of 11 days (20). A 19-year-old ALK-positive male, following BFM90 ALCL protocol, presented a survival period of 1 month (21). Moreover, it has been reported that CD56 acts as an independent prognostic factor in ALCL, and the expression of CD56 in ALCL is suggestive of a poorer overall prognosis in both the ALK-positive and ALK-negative subgroups (22). In this case, we reported an exceedingly rare ALK-negative ALCL with CD56-positive and cerebellar origin. The patient, whose initial symptoms were cephalalgia and pyrexia, evolved rapidly into a state of progressive encephalopathy. These clinical presentations are associated with PCNSTL, albeit they are devoid of characteristic stigmata. While the patient was in a comatose state and at the brink of mortality, we compassionately implemented HD-MTX based chemotherapy, followed by sequential CD30 and CD7 targeting CAR-T cell therapy. To date, the patient has survived beyond one year and is capable of independently undertaking rehabilitation therapy with the aid of mechanical apparatuses.

### Diagnostic challenges and clinical implications

PCNSALCL is an extremely rare entity in the clinical arena and is often erroneously diagnosed as meningeal infectious pathologies, whereas only 11 cases of ALK-negative PCNSALCL have been precisely recorded in the extant medical literature (14). However, the clinical presentations and imaging modalities of these rare instances exhibit significant heterogeneity among themselves, consequently exacerbating the diagnostic conundrum of this particular disease. The concomitant employment of MRI and CT modalities bears considerable import in the diagnostic workup of PCNSL (3). In line with prior case reports, the head CT scan of this patient revealed low-density lesions as the disease progressed (23). This diagnostic challenge is compounded by MRI features that often mimic inflammatory processes: approximately one-third of ALCL cases defined by intense perigyrus and leptomeningeal enhancement resembling meningitis or encephalitis, as seen in our patient (24–26). At presentation, brain MRI and DWI were unremarkable. With recurrent and worsening headache, follow-up MRI revealed patchy foci in the left cerebellar white matter, showing slightly hyperintense signals on T1/T2-weighted sequences and hyperintensity on FLAIR—findings consistent with inflammatory or infectious etiologies as reported previously. Despite empiric anti-infective therapy, the patient’s condition deteriorated.

The laboratory test results of PCNSTL patients were non-specific. CSF cytology in conjunction with flow cytometry can be utilized to confirm the presence of tumor cells (3). During the onset and treatment process of this patient, the CSF nucleated cells were found, but the CSF flow cytometry detection was not performed, which might lead to a delay in its diagnosis. Nevertheless, considering the relatively low probability of detecting tumor cells in CSF cytology, brain biopsy still represents the gold standard for diagnosis. PCNSALCL is seldom accurately diagnosed during its incipient phase. Indeed, it demands roughly 40 days to arrive at a conclusive diagnosis on average in accordance with a previous report (27). We recommend that patients presenting with headache, hemiplegia, paresthesia, aphasia, dysarthria, ataxia, hypomnesia, blurred vision, diplopia, back pain, bilateral lower limb weakness and sensory level disorder, particularly those in whom nucleated cells have been identified within the CSF, should be routinely subjected to CSF flow cytometry analysis so as to further augment the rate of early diagnosis of PCNSL. Furthermore, in respect to patients among whom a diagnosis of PCNSL fails to be ascertained via CSF assay, the reiteration of such testing may potentially bestow certain advantages, nevertheless, if a diagnosis remains elusive, a biopsy might need to be contemplated.

### Innovative treatment strategy: sequential CD30/CD7 CAR-T cell therapy

In recent years, CAR-T cell therapy has revolutionized the treatment paradigm of hematological malignancies, including CNSL, T-cell lymphoma and CD30-positive lymphoma (13). Sylvain Choquet et al. analyzed the French LOC network and reported that CD19 CAR-T cell therapy demonstrates significant efficacy in patients with relapsed or refractory PCNSL (R/R-PCNSL) (9). Significantly, the percentage of patients achieving complete remission (CR) attains a level as high as 86%. In addition, another study reported (28) that 18 patients, who were diagnosed with PCNSL and SCNSL and had experienced relapse following at least one prior systemic treatment targeting the CNS, were intravenously infused with Axicabtagene Ciloleucel. With a median 24-month follow-up, the overall response rate (ORR) was 94%, CR/undetected CR rate was 67%. However, treating T-cell malignancies with CAR-T therapy is more complex due to the similarity between normal and malignant T cells, leading to issues like fratricide.

Autologous CAR-T cell therapy, a promising treatment for T cell-derived malignancies, is widely used in clinical trials with notable efficacy. Theoretically, it is able to survive within the organism for a long time. Consequently, it can effectively inhibit disease recurrence. CD30 and CD7 have emerged as promising targets for T-cell lymphoma (10). CD30 CAR-T therapy has been effective in CD30-positive, high-recurrence lymphomas but less so in T-cell lymphoma, with high relapse rates (29). In contrast, CD7 CAR-T therapy has demonstrated significant efficacy in relapsed/refractory (R/R) T-cell lymphoma, with manageable CRS (12), the ORR was 100% (10/10), 9 patients attained CR (9/10), and 1 Monomorphic epitheliotropic intestinal T-cell lymphoma patient achieved partial remission (PR) (1/10). Another research (11) has documented that 5 CD7-positive R/R Peripheral T-cell Lymphoma patients are likely to achieve a good CR rate and a favorable safety profile after receiving CAR-T cell therapy targeting CD7, at a median of 34 days after CAR-T cell infusion, 3 patients (60%) achieved CR, 1 patient achieved PR, and 1 patient had no response, the median observation time was 234 days (range: 42–474 days), among the 3 patients who achieved CR, 2 received consolidative allogeneic hematopoietic stem cell transplantation, one of them remained progression-free at day 216, while the other relapsed at day 248 and died of chronic graft-versus-host disease on day 474. Our patient received sequential CD30 and CD7 CAR-T cell therapy. Although the initial expansion of CAR-T cells was suboptimal, subsequent infusions led to favorable expansion and significant improvement in consciousness, as evidenced by the increase in the GCS score from 5-9 to 12-15. After 15 months of follow-up, the patient remains alive, can perform rehabilitation independently, and shows no signs of tumor recurrence. This suggests that sequential CAR-T therapy targeting CD30 and CD7 may be a viable treatment option for PCNSALCL.

### Mechanistic insights and safety considerations

Despite evidence that neurotoxicity associated with CAR-T therapy in CNS lymphoma can be managed, the fundamental mechanisms of ICANS linked to CAR-T treatment remain unclear. In patients with PCNSL, CAR-T cells are capable of successful expansion and subsequent delivery to the central nervous system, where they exert their antitumor functions (30). However, the passage of CAR-T cells across the blood-brain barrier (BBB) into the CNS may also precipitate adverse events, with ICANS being a notable example (31). Available data indicate that CAR-T therapy for CNS lymphoma does not increase the likelihood of developing ICANS and can be administered safely (32). Nevertheless, the critical biological elements that govern the CNS’s tolerance to CAR-T activity have yet to be determined. Future research efforts should focus on elucidating these mechanisms to enhance treatment safety. Moreover, although autologous CAR-T cell therapy is theoretically expected to offer sustained protection against disease recurrence, its long-term implications for the immune system necessitate further exploration. Notably, the lack of ICANS in our case supports the safety profile of sequential CD30/CD7 CAR-T cell therapy for PCNSALCL.

### Cerebellar involvement: unique clinical and prognostic implications

Cerebellar lesions can trigger a diverse range of clinical manifestations, including deficits in motor coordination, impairments in balance function, abnormal eye movements, speech disorders, as well as diminished muscle tone. Up to now, there exists no curative treatment modality available for non-hereditary degenerative cerebellar ataxia. A case report (33) showed that a spinal cerebellar ataxia patient with a deteriorating condition under drug treatment received allogeneic bone marrow-derived mesenchymal stem cell (MSC) therapy and had significant improvement within 10 months post-treatment. No adverse events were reported. The enhanced Korean version of the Scale for the Assessment and Rating of Ataxia score implies MSC’s neuroprotective effect, suggesting stem cell treatment as a potential option for degenerative cerebellar ataxia. Regrettably, our patient in tumor remission had an ataxia-induced fall and subsequent cerebral hemorrhage, prompting consideration of his current state. In the foreseeable future, we eagerly anticipate the emergence of more highly promising and therapeutically effective research on cerebellar pathologies.

In conclusion, the sequential CD30 and CD7 targeting CAR-T cell therapy is a new treatment option for patients with PCNSALCL, and it is also an effective and safe treatment method. For patients with cerebellar ALCL, whether sequential CAR-T cell therapy targeting CD30 and CD7 has long-term efficacy and whether it affects the patients’ immune function both require long-term follow-up to evaluate its effectiveness and safety. As far as we know, we have reported the first successful case of ALK-negative PCNSALCL treated with CAR-T cell therapy. This case illustrates the feasibility of CD30/CD7 CAR-T therapy in CNS ALK-negative ALCL and supports further investigation into antigen-specific CAR-T approaches for rare T-cell malignancies of the CNS.

## Data Availability

The raw data supporting the conclusions of this article will be made available by the authors, without undue reservation. Further inquiries can be directed to the corresponding authors. Requests to access the datasets should be directed to Jin Rao, raojin720325@163.com.
