# Correction: Case Report: CAR-T therapy for primary cerebellar ALK-negative anaplastic large cell lymphoma

**DOI:** 10.3389/fimmu.2025.1674954

**Published:** 2025-08-22

**Authors:** Zhongqing Zou, Sihan Lai, Hai Yi, Qian Zhang, Hao Yao, Hui Yang, Ling Zhang, Alex H. Chang, Yi Su, Jin Rao

**Affiliations:** ^1^ Department of Hematology, Clinical Medical College & Affiliated Hospital of Chengdu University, Chengdu University, Chengdu, China; ^2^ Department of Hematology, The General Hospital of Western Theater Command, Sichuan Clinical Research Center for Hematological Disease, Branch of National Clinical Research Center for Hematological Disease, Chengdu, China; ^3^ Engineering Research Center of Gene Technology, Ministry of Education, Institute of Genetics, School of Life Sciences, Fudan University, Shanghai, China; ^4^ Shanghai YaKe Biotechnology Ltd., Shanghai, China

**Keywords:** primary central nervous system lymphoma (PCNSL), T-cell lymphoma, cerebellar, ALK, ALCL, CAR-T, CD30, CD7

File **Table 1** was erroneously published with the original version of this paper. The file has now been removed.

The original version of this article has been updated.

