# Supplementary material for: Case Report: CAR-T therapy for primary cerebellar ALK-negative anaplastic large cell lymphoma
Source: Front Immunol. 2025 Jul 24;16:1570214. doi: 10.3389/fimmu.2025.1570214 (PMC12328346; doi:10.3389/fimmu.2025.1570214)
Supplement: Supplementary file 1 [file Image1.pdf]

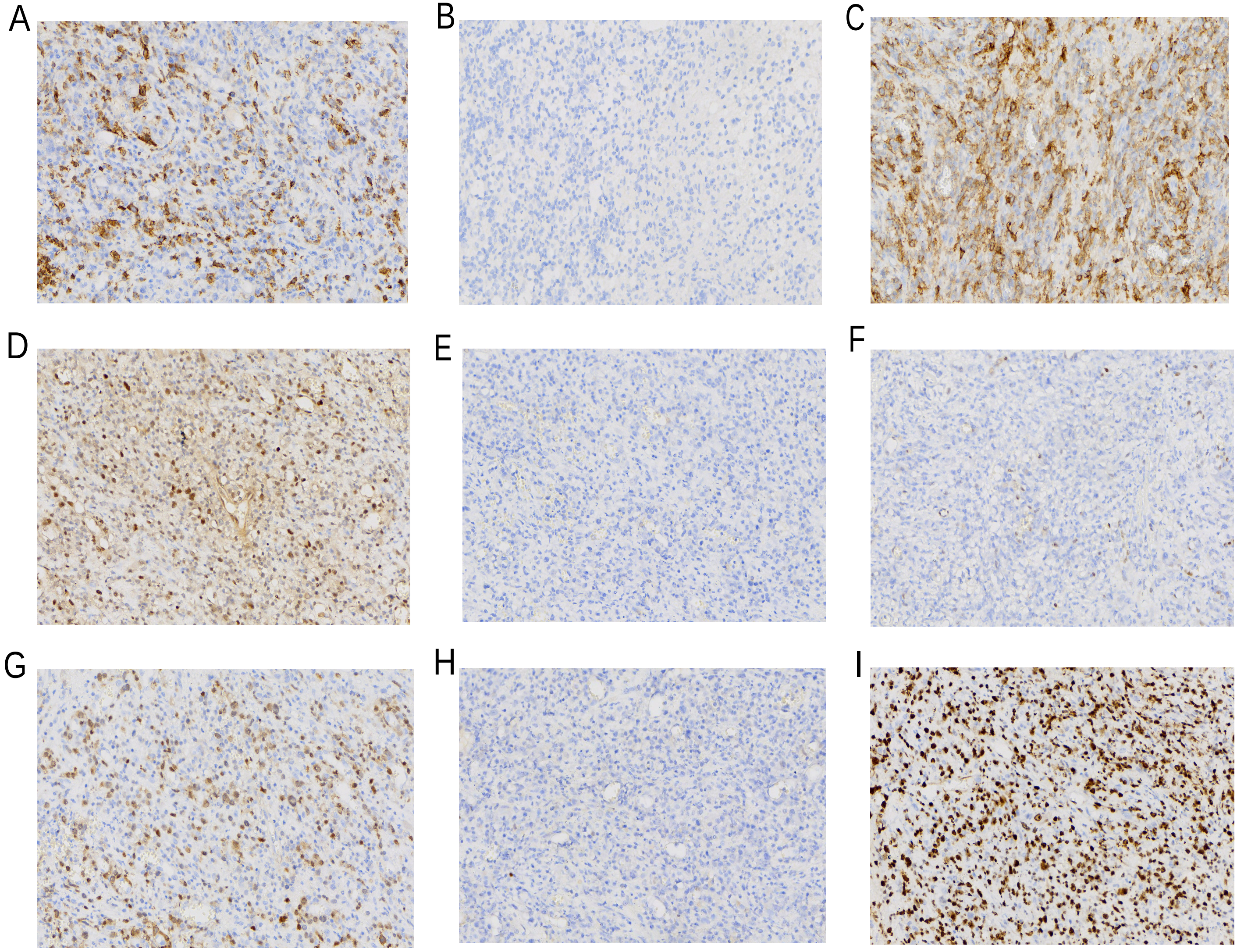

Supplementary material (Histopathology): The post-operative cerebellar pathological diagnosis is cytotoxic T-cell lymphoma, and considered as anaplastic large cell lymphoma. The immunohistochemistry indicated CD8 negative **(A, 20×),** CD20 negative **(B, 20×),** CD43 positive **(C, 20×),** C-MYC positive **(D, 20×),** PD-1 negative **(E, 20×),** LEF-1 negative **(F, 20×),** MUM-1 positive**(G 20×),** P63 negative **(H 20×),** KI-67 positive 60-70%**(I, 20×).**
